# Machine Learning Algorithms Based on Time Series Pre-Clustering for Nocturnal Glucose Prediction in People with Type 1 Diabetes

**DOI:** 10.3390/diagnostics14212427

**Published:** 2024-10-30

**Authors:** Danil E. Kladov, Vladimir B. Berikov, Julia F. Semenova, Vadim V. Klimontov

**Affiliations:** 1 Laboratory of Endocrinology, Research Institute of Clinical and Experimental Lymphology, Branch of the Institute of Cytology and Genetics, Siberian Branch of Russian Academy of Sciences (RICEL—Branch of IC&G SB RAS), 630060 Novosibirsk, Russia; d.kladov@g.nsu.ru (D.E.K.); berikov@math.nsc.ru (V.B.B.); ekmxtyjr@yandex.ru (J.F.S.); 2 Laboratory of Data Analysis, Sobolev Institute of Mathematics, Siberian Branch of Russian Academy of Sciences, 630090 Novosibirsk, Russia

**Keywords:** type 1 diabetes, continuous glucose monitoring, glucose prediction, nocturnal hypoglycemia, cluster analysis, machine learning, random forest, gradient boosting trees

## Abstract

**Background**: Machine learning offers new options for glucose prediction and real-time glucose management. The aim of this study was to develop a machine learning-based algorithm that takes into account glucose dynamics patterns for predicting nocturnal glucose in individuals with type 1 diabetes. **Methods**: To identify glucose patterns, we applied a hierarchical clustering algorithm to real-time continuous glucose monitoring data obtained from 570 adult patients. Machine learning algorithms with or without pre-clustering were used for modeling. **Results**: Eight clusters without nocturnal hypoglycemia and six clusters with at least one low-glucose episode were identified by the cluster analysis. When forecasting time series without hypoglycemia with a prediction horizon (PH) of 15 or 30 min, gradient boosting trees (GBTs) with pre-clustering and random forest (RF) with pre-clustering outperformed algorithms based on medoids of time series clusters, the Holt model, and GBTs without pre-clustering. When forecasting time series with low-glucose episodes, a model based on the pre-clustering and GBTs provided the highest predictive accuracy at PH = 15 min, and a model based on RF with pre-clustering was the best at PH = 30 min. **Conclusions**: The results indicate that the clustering of glucose dynamics can enhance the efficacy of machine learning algorithms used for glucose prediction.

## 1. Introduction

Type 1 diabetes (T1D) remains a major challenge for people living with the disease and for health care systems. According to a recent report from the International Diabetes Federation, 8.75 million people worldwide are living with T1D; in 2022, the number of diabetes-related deaths was estimated at 182,000 [1].

The development and implementation of precision-medicine approaches to diagnosis, prevention, and treatment are expected to reduce the diabetes burden [2,3]. Particular hopes are associated with technological solutions such as closed-loop automated insulin delivery and next-generation continuous glucose monitoring (CGM) systems [4,5]. However, these technologies are not yet available to most T1D patients. Regardless of the method of insulin delivery, accurate prediction of glucose level during the action of exogenous insulin is essential for correct dosing and, therefore, for improving the safety and efficacy of the treatment.

Glycemic management at night presents a particular challenge for people with diabetes and clinicians. First of all, this is associated with the risk of nocturnal hypoglycemia (NH), which is especially high in patients with T1D on basal bolus insulin therapy [6]. Although this complication often goes unrecognized, it can be a trigger for severe cardiovascular events, including “death-in-bed” syndrome, and can affect quality of life, mood, and work performance the following day [7]. The awakening response to NH is impaired in people with T1D [8]. Advances in technology, such as CGM and automated insulin delivery, are expected to significantly reduce the burden of hypoglycemia in patients with T1D [9]. In this regard, accurate glucose prediction is becoming increasingly important.

In recent years, a data-driven approach for glucose prediction has shown significant progress. This approach is based on machine learning (ML) algorithms trained on large datasets of glucose measurements typically accumulated from CGM. In most of the proposed models, the prediction horizon (PH) varied from 15 to 60 min as a compromise between predictive accuracy and the time required to take action to prevent an adverse glycemic event. To date, a number of ML algorithms have been tested for glucose levels or hypoglycemia prediction. Among them, artificial neural networks, random forest (RF), support vector regression, decision trees, and autoregressive models were the most commonly used. In addition, deep learning algorithms and assembled ML were tested to increase the predictive performance [10,11,12,13]. Some ML models operating with CGM data and clinical parameters or data from other devices have also been proposed [14,15,16].

The complexity of glucose dynamics makes it difficult to achieve a universal model of glucose prediction. The proposed ML algorithms perform well when predicting a single time series; however, the results for multiple time series may be worse since algorithms do not take into account the data structure. We proposed that the performance of ML algorithms can be improved by pre-clustering the glucose time series that turn them into several more homogeneous datasets. In this approach, forecasting is performed within a specific glucose cluster. Recently, we applied hierarchical clustering algorithm to assess the cluster structure of nocturnal CGM data from people with T1D managed with multiple daily insulin injections. Ten clusters without episodes of low glucose and six clusters with the episodes were identified [17].

In this study, we developed ensemble forecasting models based on the clustering of CGM data and ML algorithms to predict nocturnal glucose levels in patients with T1D.

## 2. Materials and Methods

### 2.1. Dataset

A dataset of the real-time CGM recordings from an institutional database was used for developing and testing the glucose prediction algorithms. This database was registered by the Federal Service for Intellectual Property (certificate 2023623235 dated 26 September 2023). Current acute hyperglycemia crises, pregnancy, end-stage renal disease, and severe accompanied diseases were considered as principal exclusion criteria.

We analyzed CGM data obtained from 570 patients with T1D: 211 men and 359 women aged from 18 to 73 years (median 36 years). Overall, 345 patients had a body mass index less than 25 kg/m^2^, 142 were overweight, and 82 patients had obesity. Diabetes duration varied from 0.5 to 55 years (median 16 years). The following diabetic complications and comorbidities were identified: diabetic neuropathy (*N* = 421), chronic kidney disease (*N* = 370), diabetic retinopathy (*N* = 317), arterial hypertension (*N* = 217), metabolic-associated fatty liver disease (*N* = 202), peripheral artery disease (*N* = 85), diabetic foot (*N* = 44), and coronary artery disease (*N* = 29).

Patients were managed with multiple daily injections (*N* = 395) or continuous subcutaneous insulin infusion (*N* = 175) with insulin analogues. Mean daily insulin dose was 0.68 IU/kg (range: 0.2–2.0 IU/kg). The levels of glycated hemoglobin A1c was 8.1%, i.e., 65 mmol/mol (range: 4.7–15.2%, i.e., 28–143 mmol/mol).

The CGM was performed with Medtronic Paradigm MMT-722, MiniMed Paradigm Veo (MMT-754), and CareLink™ software (2.5.524.0, v. 2.5A, Medtronic, Minneapolis, MN, USA). The insulin pumps Medtronic Paradigm MMT-722 and MiniMed Paradigm Veo (MMT-754) were used for insulin infusion. The CGM duration varied from 3 to 14 days, with a median of 6 days.

### 2.2. Data Preprocessing

We extracted nocturnal (0.00–5.59) segments with a length of 72 measurements from each CGM record. Segments with a gap of three or more consecutive values and segments with more than 10% of missing values were excluded. Single and double missing values were filled with means of closest left and right non-zero values. No feature engineering was performed. A time series was represented as a vector in $R^{n}$, where n is the length of the time series.

All the time series were divided into two groups depending on the presence of nocturnal hypoglycemia (NH) episode(s). The episode was defined as a level of interstitial glucose < 3.9 mmol/L (<70 mg/dL) that persisted at least 15 min. Finally, 3318 time series were included in analysis (Table 1).

As the next step, the set of time series with and without NH was further divided into training, validation, and test samples in a ratio of 0.7:0.1:0.2. So, each object in the dataset represents a patient with a corresponding vector of glucose values.

### 2.3. Algorithms

#### 2.3.1. Clustering Procedure

Cluster analysis is the basis of the proposed predictive algorithms. We applied clustering to obtain more homogeneous sets of data for further processing by ML algorithms. A number of algorithms were proposed for clustering time series [18]. Taking into account the short length of the analyzed time series and the large degree of glucose variability, we chose a hierarchical clustering algorithm that can determine the structural properties of data, and the obtained results are therefore more interpretable. Interpretability was assessed by further expert analysis. The number of clusters was chosen empirically based on the Silhouette Score [19], visualization of obtained solutions, and expert assessment.

The Ward’s method was chosen as the criterion responsible for the merging classes [20]. The Ward algorithm in hierarchical clustering works by iteratively merging the two clusters that result in a minimum increase of the total within-cluster variance after merging. This process continues until a predefined number of clusters is reached.

#### 2.3.2. Forecasting Using Medoids of Time Series Clusters (MTSCs)

Let $t_{1}^{\prime },\ldots ,t_{n}^{\prime }$ be a set of training time series of a length N; $t_{1},\ldots ,t_{m}$ is a set of test time series of length N; and h is a forecasting horizon. Next, we consider the clustering algorithm.

An algorithm consists of several steps:

- Apply clustering algorithm for $t_{1}^{\prime },\ldots ,t_{n}^{\prime }$ and find the optimal number of clusters $C_{1},\ldots ,C_{k}$;
- Evaluate the obtained clustering structure;
- For each cluster, specify a typical object (centroid, medoid, etc.) $c_{1},\ldots ,c_{k}$;
- For $t_{i},i=1,\ldots ,m$, find the nearest (in the sense of a given distance) typical object $c_{j}:\rho \left(t_{i},c_{j}\right)\leq \rho \left(t_{i},c_{p}\right),p=1,\ldots ,k$, where ρ(·,·) is a metric function between observations;
- As a prediction for $t_{i}$, take the next h components of the selected typical object $c_{j}$.

We applied a Monte Carlo algorithm for evaluation of the clustering structure, as described earlier [21]. In this study, we considered the medoid of each cluster as a typical observation.

This study represents a naive realization of the proposed algorithm, and it does have several drawbacks. Firstly, for forecasting, this algorithm takes into account only one typical object, so if the nearest typical object is not really close to the predicted time series, the forecast may be inaccurate. Secondly, the algorithm does not consider a trend of the predicted time series and its structure because the algorithm depends only on the distance between the time series.

#### 2.3.3. Forecasting Using Weighted Medoids of Time Series Clusters (WMTSCs)

In this paper, we propose one more prediction algorithm based on the same idea but with a different heuristic. This is a modification of the previous algorithm that takes into account all typical objects selected at the clustering stage when forecasting. This can be done by taking into consideration the weights or probabilities of the predicted time series belonging to the clusters.

For a final forecast, we take weighted forecasts of all typical objects. As mentioned above, let $t=(t_{1},\ldots ,t_{N})$ be a predicted time series; $C=\{c^{1},\ldots ,c^{k}\}$ are objects typical of each cluster (for the convenience, the indexes are now located on top); h is a forecasting horizon; ρ(·,·) is a metric function between objects. It is possible to define the distance vector between the predicted time series and a set of typical objects: $d^{\prime }=\rho \left(t,C\right)=(d_{1}^{\prime },\ldots ,d_{k}^{\prime })$, where $d_{i}^{\prime }=\rho (t,c^{i})$. We also consider the inverse of the vector $d^{\prime }:$ vector $d=\left(\frac{1}{d_{1}^{\prime }},\ldots ,\frac{1}{d_{c}^{\prime }}\right)$. We define the weights as follows: $\omega =\left(\omega_{1},\ldots ,\omega_{c}\right)=softmax(d)$, where $softmax\left(x_{1},\ldots ,x_{n}\right)=\left(\frac{e^{x_{1}}}{\sum_{i}e^{x_{i}}},\ldots ,\frac{e^{x_{n}}}{\sum_{i}e^{x_{i}}}\right)$. Consequently, as a final forecast, we propose weighted sums of forecasts of individual typical objects:

$$\left({\hat{t}}_{N+1},\ldots ,{\hat{t}}_{N+h}\right)=\left(\omega_{1},\ldots ,\omega_{c}\right)\cdot \left[\begin{array}{ccc} c_{N+1}^{1} & \cdots & c_{N+h}^{1}\\ \vdots & \ddots & \vdots \\ c_{N+1}^{k} & \cdots & c_{N+h}^{k} \end{array}\right]=\left(\sum_{i=1}^{k}\omega_{i}c_{k+1}^{i},\ldots ,\sum_{i=1}^{c}\omega_{i}c_{N+h}^{i}\right).$$

The weighted forecast algorithm is the same as the naive algorithm except the final step. This version of algorithm is no longer dependent on only one typical object.

#### 2.3.4. Combined Algorithms Based on the Cluster Analysis and Supervised ML

One more modification of the medoid forecast algorithm involved ML models for the forecasting. While the previous algorithms used only medoids values as the aggregation values over a cluster, this version of the algorithm takes each observation in a cluster to make a prediction.

The algorithm consists of several steps:

1. Apply clustering algorithm for $t_{1}^{\prime },\ldots ,t_{n}^{\prime }$ and find the optimal number of clusters $C_{1},\ldots ,C_{k}$;
2. Evaluate the obtained clustering structure;
3. For each cluster $C_{1},\ldots ,C_{k}$, train ML model for prediction at the determined prediction horizon. Cluster $C_{i}$ is a training dataset for ${model}_{i}$;
4. For each cluster $C_{1},\ldots ,C_{k}$, specify a typical observation (centroid, medoid, etc.) $c_{1},\ldots ,c_{k}$;
5. For $t_{i},i=1,\ldots ,m$, find the nearest cluster, i.e., the nearest typical object $c_{j}:\rho \left(t_{i},c_{j}\right)\leq \rho \left(t_{i},c_{p}\right),p=1,\ldots ,k$, where ρ(·,·) is a metric function between objects;
6. Predict $t_{i}$ with a model trained on the nearest cluster found in step 5.

#### 2.3.5. Baseline Algorithms

We used the Holt model and ensemble algorithms based on decision trees as baselines. We applied the Holt model, which takes into account the trend of the time series and uses exponential smoothing. Due to the length and specificity of the time series considered in this study, there is no seasonality in them. We applied ensemble algorithms because they are among the best machine learning algorithms to date, including for the time series forecasting problem.

We also used two types of ensemble algorithms:

- An algorithm that uses averaging to improve the predictive accuracy. In this study, we used an RF algorithm. In the RF, each tree in the ensemble is built from a sample drawn with replacement (i.e., a bootstrap sample) from a training set. This algorithm takes the average prediction of each tree in the ensemble as a result;
- An algorithm that builds an additive model in a forward stage-wise fashion. In each stage, a regression tree is fit onto the negative gradient of the given loss function to minimize it. We used a gradient boosting trees (GBTs) model.

### 2.4. Assessment of the Performance of Algorithms

For the assessment of the algorithms, the following metrics were calculated:

- Root Mean Squared Error (RMSE): $\sqrt{\frac{\sum_{i=1}^{N}{\left(t_{i}-{\hat{t}}_{i}\right)}^{2}}{N}}$;
- Mean Absolute Error (MAE): $\frac{\sum_{i=1}^{N}\left|t_{i}-{\hat{t}}_{i}\right|}{N}$;
- Mean Absolute Percentage Error (MAPE): $\sum_{i=1}^{N}\frac{\left|t_{i}-{\hat{t}}_{i}\right|}{\left|t_{i}\right|}$.

Here, $t_{i}$ is a true value of the time series, and ${\hat{t}}_{i}$ is a predicted value.

Metrics for all algorithms were calculated for 15 min and 30 min PH.

### 2.5. Coding

We used PyCharm for coding and Jupiter Notebook (JetBrains, Prague, Czech Republic) to visualize the solutions. The programming language was Python v. 2020.3.3 (Python Software Foundation, Wilmington, Delaware, DE, USA). Stack: numpy, pandas, sklearn, matplotlib libraries.

## 3. Results

### 3.1. Clusters of Nocturnal Glucose Dynamics

**Figure 1:**
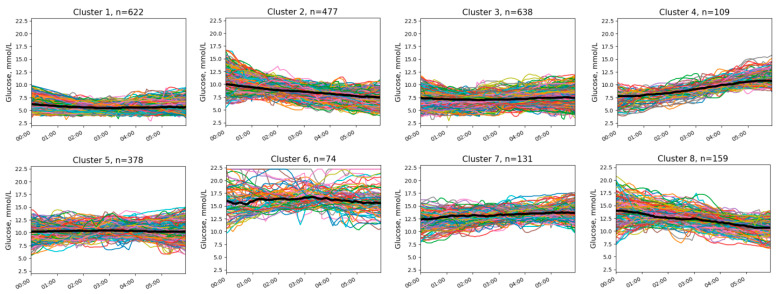
Clusters of nocturnal glucose dynamics without episodes of NH in patients with T1D. Different colors indicate individual glucose level profiles.

**Figure 2:**
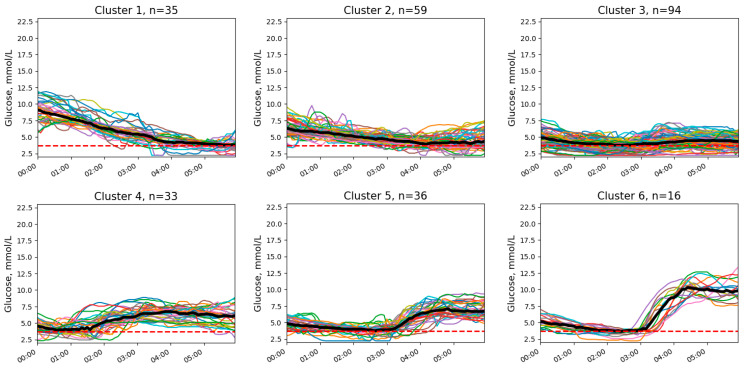
Clusters of nocturnal glucose dynamics with episodes of NH in patients with T1D. The red line represents the hypoglycemia threshold (3.9 mmol/L). Different colors indicate individual glucose level profiles.

We identified eight clusters without NH (Figure 1) and six clusters with at least one NH episode (Figure 2).

Clusters differed from each other in initial and final glucose levels and the presence of trends, either downward or upward. In addition, clusters with NH differed in the timing of low-glucose episodes and in the presence or absence of an upward trend after the episode.

The two most common patterns without NH (cluster 1 and 3; Figure 1) demonstrated stable and target (or near target) glucose levels throughout the night. Other patterns included elevated glucose levels at different times of the night. Hypoglycemic patterns were typically characterized by midnight glucose levels slightly above the hypoglycemic threshold (clusters 3–6; Figure 2). Two patterns (clusters 1 and 2; Figure 2) demonstrated normal or even elevated initial glucose levels but had a pronounced downward trend.

**Figure 3:**
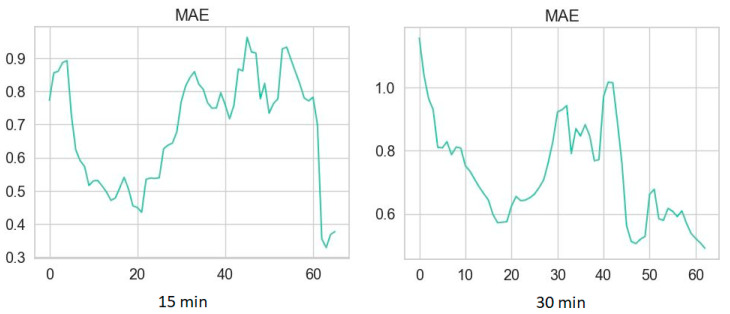
Dependence between MAE and length of predicted time series without NH.

**Figure 4:**
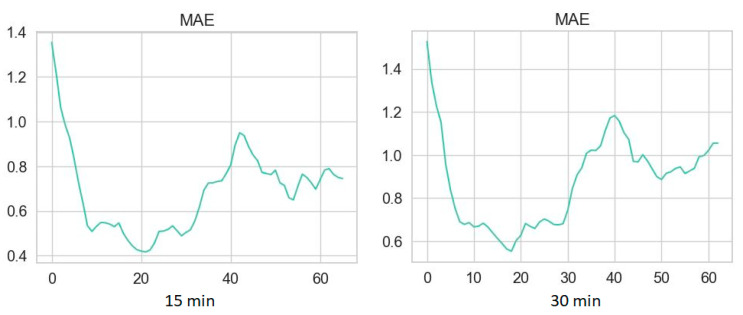
Dependence between MAE and length of predicted time series with NH.

Plots of MAE are presented in Figure 3 and Figure 4 for time series without NH episodes and for those with NH, respectively.

### 3.2. Performance Metrics of Glucose Prediction Algorithms

Metrics of the algorithms operating with CGM data without NH are presented in Table 2. In the models with 15 min PH, GBTs with pre-clustering provided the lowest MAE and MAPE values; meanwhile, RF with clustering provided the lowest RMSE. At 30 min PH, GBTs with clustering outperformed other models, providing the minimal MAE and MAPE.

When forecasting time series with low-glucose episodes, models based on the clustering and GBTs provided the highest predictive accuracy at PH = 15 min (Table 3). However, the model based on RF with pre-clustering outperformed the other models, with PH = 30 min.

## 4. Discussion

Algorithms of ML trained on real-time CGM data have opened up new possibilities for “on-the-fly” glucose prediction. To date, many algorithms have been proposed for this task, but an ideal model still does not exist. To increase the reliability of the forecast, designers choose different strategies. One strategy is to combine glucose data with other types of data, such as carbohydrates intake, insulin doses, physical activity, heart rate, breath samples, electrocardiogram or electroencephalogram parameters, galvanic skin response, body temperature, etc. [22]. Other researchers have implemented various CGM metrics, including glucose variability parameters, to improve the accuracy of their models [15,23]. Recently, a number of deep learning architectures [24,25,26,27,28], meta-learning [24], and transfer learning [29] have been tested for glucose prediction, with some promising results. In addition, ensemble learning has been proposed to improve the performance of the models [30,31,32]. Although the lack of validation studies is still a major problem, some of the proposed approaches have been demonstrated to be clinically useful [29,33].

The heterogeneity of glucose dynamics is a challenge in glucose forecasting. T1D is increasingly described as a heterogeneous disease with different endotypes [34,35] and glycemic phenotypes [36]. Glucose dynamics may also vary inter- and intra-individually depending on endogenous and behavioral factors, nutrition, and insulin therapy. Some patients exhibit glucose dynamics that are particularly difficult to predict [37]. Taking this into account, a personalized approach for glucose prediction, which involves training ML algorithms on data from a specific patient instead of population data, has been proposed. However, the performance of this approach is highly dependent on the amount and quality of the data available for a specific individual [38]. Another idea is to identify patterns of glucose dynamics and implement these patterns into predictive models. Previously, we identified different patterns of glucose dynamics in T1D patients using hierarchical cluster analysis [17]. In the present study, we aimed to develop an algorithm for glucose prediction that would take into accounts these glucose dynamics patterns.

The principal idea of the proposed approach is to cluster the time series of glucose values derived from CGM so that an ML algorithm can further operate with a time series representing a particular pattern of glucose dynamics. As far as we know, such an approach for glucose prediction was developed herein for the first time. We demonstrated that a combination of cluster analysis, which is an unsupervised ML technique, with supervised ML algorithms (RF or GBTs) provided an increase in the quality of nocturnal glucose prediction in people with T1D when compared to the supervised or unsupervised ML alone. Specifically, pre-clustering with RF or GBTs outperformed algorithms based on MTSCs and WMTSCs, the Holt model, and GBTs without pre-clustering.

The developed algorithms predict glucose levels at a PH of 15 and 30 min. The selection of PH was based on both clinical and computational considerations. A time advantage of 15 min or especially 30 min opens a window of opportunity to prevent an episode of hypoglycemia or hyperglycemia by ingesting carbohydrates, changing the insulin delivery rate, or giving a correction bolus. Further increases in the PH usually result in a decrease in the accuracy of the prediction [13]. Therefore, 15 min and 30 min PH sizes are the most commonly chosen for short-term glucose forecasting [22].

The approach we developed can integrate any ML techniques, including deep learning algorithms. Despite this, our approach has some limitations. First of all, it assumes the presence of patterns in the training sample. If there are none, a model may perform even worse than other algorithms for time series prediction. A set of training time series is required. Since our approach involves cluster analysis, it is not suitable for predicting a single time series. The algorithm cannot reliably predict glucose values that are above the maximum and below the minimum value in the training set. The latter, in turn, may depend on the detectable range of a CGM system.

Ours is a proof-of-concept study. In future research, it would be useful to determine which ML algorithms can provide a more accurate forecast within the proposed concept as well as to estimate the effectiveness of the models prospectively in a validation study.

## 5. Conclusions

In this study, we developed a new approach to glucose prediction in people with T1D based on the combination of supervised and unsupervised ML. The algorithms we generated combine a hierarchical cluster analysis of real CGM data (unsupervised learning) with supervised ML techniques: RF and GBTs. The pre-clustering procedure ensures that the algorithm switches to forecasting within a specific pattern of glucose dynamics, which increases the predictive accuracy. The approach we developed can be used in future research for developing decision-support systems or automated insulin delivery systems for patients with T1D.

## Figures and Tables

**Table 1:** Number of analyzed time series extracted from CGM data.

Parameter	Time Series Without NH	Time Series with NH
$N_{init}$	3376	449
*Delta (%)*	0.02	19.2
$N_{final}$	3318	363

$N_{init}$ = number of the time series before preprocessing; *Delta* = percentage of the excluded time series; $N_{final}$ = number of the time series after preprocessing; CGM, continuous glucose monitoring; NH, nocturnal hypoglycemia.

**Table 2:** Metrics of nocturnal glucose prediction algorithms operating with CGM data without NH episodes.

PH	Algorithm	RMSE	MAE	MAPE
15 min	MTSCs	1.643	1.221	0.148
	WMTSCs	3.555	3.029	0.434
	Holt model	0.640	0.327	0.039
	GBTs without pre-clustering	0.600	0.324	0.037
	RF with pre-clustering	**0.532**	0.322	0.038
	GBTs with pre-clustering	0.542	**0.305**	**0.034**
30 min	MTSCs	1.742	1.276	0.154
	WMTSCs	3.553	3.028	0.433
	Holt model	1.219	0.634	0.074
	GBTs without pre-clustering	**0.770**	0.450	0.052
	RF with pre-clustering	0.811	0.526	0.062
	GBTs with pre-clustering	0.771	**0.422**	**0.051**

CGM, continuous glucose monitoring; GBTs, gradient boosting trees; MAE, mean absolute error; MAPE, mean absolute percentage error; MTSCs, medoids of time series clusters; NH, nocturnal hypoglycemia; PH, prediction horizon; RF, random forest; RMSE, root mean squared error; WMTSCs, weighted medoids of time series clusters. The smallest error values are highlighted in bold.

**Table 3:** Metrics of nocturnal glucose prediction algorithms operating with CGM data with NH episodes.

PH	Algorithm	RMSE	MAE	MAPE
15 min	MTSCs	1.916	1.406	0.274
	WMTSCs	2.706	2.193	0.457
	Holt model	0.797	0.440	0.086
	GBTs without clustering	1.169	0.724	0.120
	RF with pre-clustering	0.755	0.488	0.091
	GBTs with pre-clustering	**0.682**	**0.451**	**0.080**
30 min	MTSCs	1.936	1.433	0.278
	WMTSCs	2.706	2.194	0.457
	Holt model	1.461	0.890	0.154
	GBTs without pre-clustering	1.534	1.027	0.171
	RF with pre-clustering	**1.201**	**0.787**	**0.150**
	GBTs with pre-clustering	1.305	0.918	0.166

CGM, continuous glucose monitoring; GBTs, gradient boosting trees; MAE, mean absolute error; MAPE, mean absolute percentage error; MTSCs, medoids of time series clusters; NH, nocturnal hypoglycemia; PH, prediction horizon; RF, random forest; RMSE, root mean squared error; WMTSCs, weighted medoids of time series clusters. The smallest error values are highlighted in bold.

## Data Availability

The source data are available from the correspondence author upon reasonable request.
